# Kocuria kristinae-Induced Infective Endocarditis: Unveiling an Emerging Threat in Clinical Practice

**DOI:** 10.7759/cureus.58979

**Published:** 2024-04-25

**Authors:** Tracy-Ann Poyser, Damilola Gbadebo, Jacob Krebs, Jordan M Brock, Eric Robinson

**Affiliations:** 1 Internal Medicine, Unity Health-White County Medical Center, Searcy, USA; 2 Cardiology, Unity Health-White County Medical Center, Searcy, USA

**Keywords:** gram-positive cocci, duke's criteria, prosthetic heart valve, infective endocarditis, kocuria kristinae

## Abstract

Infective endocarditis (IE) remains a formidable challenge in clinical practice due to several causative agents, each presenting with unique diagnostic and therapeutic dilemmas. *Kocuria kristinae*, a coagulase-negative, catalase-positive Gram-positive coccus, has recently emerged as an uncommon but increasingly recognized pathogen in the cause of IE. This case report highlights the clinical characteristics, risk factors, and challenges associated with *Kocuria kristinae*-induced IE. We conducted a comprehensive literature review and identified several case reports on *Kocuria kristinae* as a causative agent. Due to its indolent nature and the subtle presentation of symptoms, along with its ability to form biofilms, delayed diagnosis of *Kocuria *is often seen, thereby emphasizing the need for heightened clinical suspicion. The predisposing factors for *Kocuria kristinae* infection include underlying cardiac abnormalities, prosthetic heart valves, and immunocompromised states.

Additionally, antimicrobial susceptibility patterns and optimal treatment strategies remain unclear, warranting further investigation. This abstract presents the case of a 75-year-old male with IE secondary to *Kocuria kristinae* on a prosthetic mitral valve. We aim to highlight the need for increased awareness among clinicians to facilitate early recognition and prompt initiation of targeted therapeutic interventions. Unraveling the intricacies of *Kocuria kristinae's* pathogenicity is crucial for refining diagnostic approaches and optimizing patient outcomes.

## Introduction

Infective endocarditis (IE) is the term that denotes a bacterial, viral, or fungal infection of the endocardial surfaces of the heart. Men older than 60 years of age with cardiac conditions (i.e., valvular heart disease, prosthetic valves, prior IE) and IV drug users (IVDU) have an increased risk of IE. Except for* Staphylococcus aureus* as an organism that can infect an intact endothelium, most infectious agents need an insult to the endothelium or a nidus such as a thrombus. 

According to an article by Mahbub et al., the United States has the highest incidence of IE, with 15 cases per 100,000 people, driven by an aging population and an increase in IVDU [1]. The diagnosis of IE is made clinically using a set of rules known as the Duke Criteria, which is made up of both major and minor criteria [2].

Major criteria 

The major criteria included positive blood cultures from two separate samples with typical organisms (viridans streptococci, *Staphylococcus aureus*, *Streptococcus gallolyticus*, HACEK group (*Haemophilus *species, *Aggregatibacter actinomycetemcomitans*, *Cardiobacterium hominis*, *Eikenella corrodens*, *Kingella kingae*), community-acquired enterococci without a primary focus) or persistently positive blood cultures with microorganisms that typically cause endocarditis from blood cultures drawn 12 hours apart, or a single positive blood culture for *Coxiella burnetii*, or an anti-phase 1 IgG titer greater than or equal to 1:800. Echocardiographic findings such as valvular vegetation, abscess, new valvular regurgitation, or prosthetic valve dehiscence are also included [2].

Minor criteria

Fever >100.4F, IVDU or predisposing heart pathology, vascular abnormalities (arterial emboli, septic infarcts, hemorrhages intracranial or conjunctival, mycotic aneurysms, or Janeway lesions), immunologic criteria, or a positive blood culture not fulfilling major criteria of an infection with a common organism are considered as minor criteria [2].

A definitive diagnosis is made if two of the major criteria are present, one major criterion is met with greater than or equal to three minor criteria, or all five minor criteria are met. Although not included in the Duke criteria, electrocardiography should be used in patients suspected of IE to assess for new conduction abnormalities such as atrioventricular (AV) blocks.

*Kocuria Kristinae* is a facultative, anaerobic, catalase-positive, coagulase-negative, and Gram-positive cocci commonly found on skin flora and oral mucosa that was first discovered in 1974 [3]. This bacterium is now being isolated in patients with IE and with bacteremia secondary to central venous catheter infections. It is speculated that the mode of virulence is through biofilm formation, although no biofilm evidence has yet been proven [4].

Biofilm is defined as microbial cells assembled and irreversibly attached to a surface, composed of extracellular polymeric substances (EPS), with a large component being polysaccharides. Extracellular polymeric substances act by hydrating the cells, along with the transportation of nutrients [5].

## Case presentation

In this case review, we present a 75-year-old man with a pertinent history of atrial fibrillation and two previous distinct diagnoses of endocarditis, both treated with porcine mitral valve replacements, who presented to the emergency department in November 2023 with generalized weakness and malaise. He reported that these symptoms felt similar to those when he had endocarditis and uncontrolled atrial fibrillation in the past. 

In April 2023, the patient presented with similar symptoms of weakness and fatigue and had been found to have methicillin-resistant* Staphylococcus aureus* (MRSA) bacteremia. A transesophageal echocardiogram (TEE) was completed during that visit, showing IE involving his porcine mitral valve, which had already been replaced. He received a repeat valve replacement with a bioprosthetic porcine valve and was placed on warfarin. 

**Figure 1:**
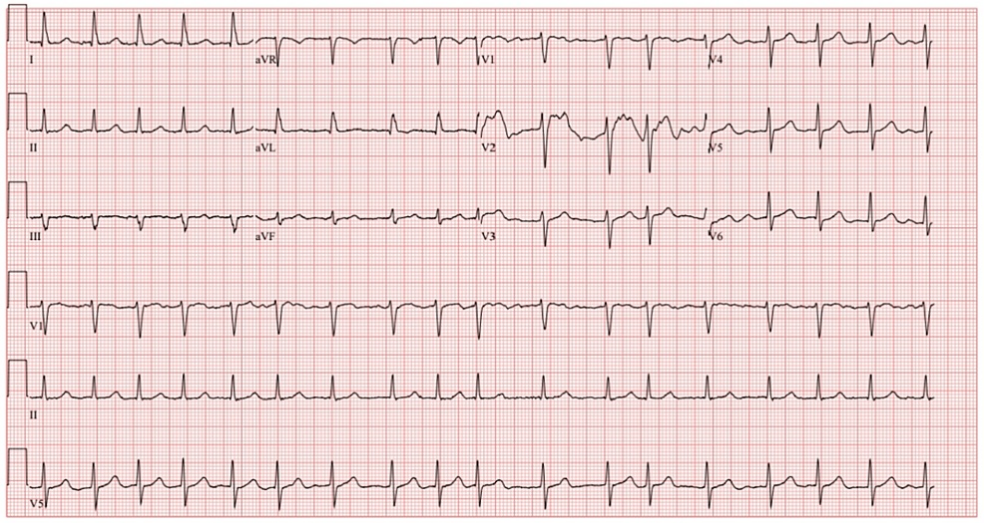
The EKG on arrival showed atrial fibrillation with RVR. EKG: electrocardiogram; RVR: rapid ventricular response

During his most recent visit and initial arrival at the emergency department, his vital signs showed a blood pressure of 95/55 mmHg, a heart rate of 129 beats per minute, a respiratory rate of 14 breaths per minute, and an oxygen saturation of 98% in ambient air. During the physical examination, he was alert and oriented to the person, place, and time. Lungs were clear to auscultation bilaterally. A cardiovascular exam noted the patient’s heart rhythm to be irregularly irregular, with a grade 2 systolic ejection murmur. His abdomen was distended but non-tender to palpation, with normal bowel sounds. He was reported to have lower extremity edema. No focal neurological deficits were observed. The initial electrocardiogram (EKG) revealed the patient to be in atrial fibrillation with rapid ventricular response (RVR) (Figure 1).

Initial laboratory results (shown in Tables 1-2) were pertinent for leukocytosis with neutrophilic predominance, elevated creatinine, elevated brain natriuretic peptide (BNP), and elevated procalcitonin. 

**Table 1:** The patient's initial complete blood count presentation

Complete blood count (CBC)		
Variable	Results on arrival	Reference range
White blood cell count	20.9	4.50-11.00 k/uL
Red blood cell count	3.68	4.40-5.90 M/uL: male; 3.80-5.20 M/uL: female
Hemoglobin	9.9	13.0-18.0 g/dL: male; 12.0 -16.0 g/dL: female
Hematocrit	30.5	40.0 -52.0%: male; 35.0-47.0%: female
Mean corpuscular volume (MCV)	83	80.0 -100.0 fL
Platelet count	150	150 -440 k/uL
Absolute neutrophil count	18.61	1.80 -7.70 k/uL
International normalized ratio (INR)	7.8	0.8-1.2 ratio

**Table 2:** The patient's initial basic metabolic panel on presentation

Basic metabolic panel (BMP)		
Variable	Results on arrival	Reference range
Sodium	138	136-145 mEq/L
Potassium	4.3	3.4-4.4 mEq/L
Chloride	106	98-107 mEq/L
Bicarbonate	20	22-29 mEq/L
Anion gap	12	8-12 mEq/L
Blood urea nitrogen (BUN)	59	8-26 mg/dL
Creatinine	2.6	0.72-1.25 mg/dL
Calcium	8.5	8.4-10.2 mg/dL
Phosphorus	3.9	2.3-4.7 mg/dL
Magnesium	2.0	1.6-2.6 mg/dL
Lactic acid	1.6	0.5-2.0 mmol/L
Brain natriuretic peptide (BNP)	667	<=100 pg/mL

**Figure 2:**
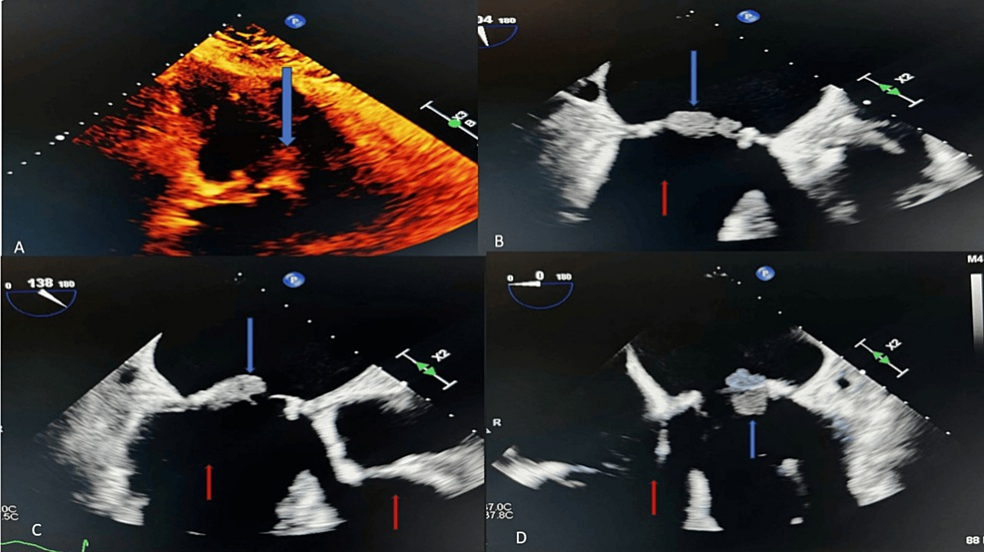
Echocardiographic image showing mobile vegetation (blue arrows) on the anterior mitral leaflet and moderately dilated right and left atriums (red arrows).

Due to his tachycardia and leukocytosis, the patient met systemic inflammatory response syndrome (SIRS) criteria and had a quick sequential organ failure assessment (qSOFA) score of one. He was given 500 mg of IV fluids as opposed to 30 milliliters/kilogram due to his history of congestive heart failure and a B-natriuretic peptide BNP of 667 on arrival. Two blood culture samples were collected, and the patient was started on empiric antibiotics with IV cefepime and IV doxycycline. Urinalysis was unremarkable. The chest X-ray was negative for any acute cardiopulmonary processes. A transthoracic echocardiogram (TTE) was completed, which showed mobile vegetation on the anterior mitral leaflet, moderately dilated right and left atrium, a bioprosthetic mitral valve with elevated gradients, trivial regurgitation, and possible vegetation (Figure 2).

Due to concerns for IE, cardiology was consulted, and a recommendation was made for a TEE.

Blood cultures returned positive for *Kocuria kristinae*. Following the guidance of our infectious disease specialists, doxycycline was discontinued, and the patient was started on vancomycin and continued on cefepime. Given the positive blood cultures and echocardiographic evidence of vegetation, the patient met diagnostic (modified Duke) criteria for definitive IE due to *Kocuria kristinae*. 

Due to ongoing persistent atrial fibrillation with a rapid ventricular response, the cardiology team recommended administering digoxin 500 mcg IV once and starting the patient on metoprolol succinate 25 mg once a day (qd). The patient continued to be hypotensive and tachycardic with leukocytosis in the setting of his bacteremia and was treated with IV fluid. He was observed to have a supratherapeutic international normalized ratio (INR) of 7.8 (Table 1) and was treated with vitamin K, with subsequent improvement in his INR. Interdisciplinary consultation recommended that the patient would benefit from another mitral valve replacement. However, after communicating with the patient’s previous cardiothoracic surgeon, it was deemed that the patient was an unlikely candidate for another mitral valve replacement due to ongoing comorbidities and prior replacements. The palliative care team was consulted, and the patient was discharged home with hospice care on ceftriaxone for six weeks. 

## Discussion

Infective endocarditis is a severe condition affecting approximately three to 10 per 100,000 individuals annually. The overall mortality rate of IE is about 25%, despite the frequent medical advances that take place every year, with higher mortality seen in Gram-negative infections [6]. One systematic review from 1990-2019 found that both the reported number of cases and deaths from IE have increased drastically, from ~478,000 cases reported in 1990 to 1,090,530 reported cases in 2019. Reported deaths from those same years ranged from 28,750 in 1990 to 66,320 in 2019. This same study also found that older patients and men were typically more severely affected by IE [6]. While the increase in the number of cases of IE over the past 30 years could be secondary to the improved ability to diagnose the disease due to medical advancements, it does not diminish the severity of the disease or the importance of timely and early diagnosis to begin treatment as soon as possible. There is also a possibility of increased prevalence due to antibiotic resistance. One critical diagnostic criterion for IE is determining whether it involves a native or prosthetic valve, which affects treatment and mortality rates.

Our patient had already had two previous episodes of IE, requiring two mitral valve replacements. His second replacement surgery was considered high-risk due to his various other comorbidities and required long discussions before his cardiothoracic surgeon finally agreed to the procedure. Prosthetic valve endocarditis (PVE) accounts for approximately 20% of IE cases. It is the most severe form of IE, as it is more frequently associated with higher morbidity and mortality when compared to native valve IE [7]. Prosthetic valve endocarditis can be further classified as early or late PVE, with early PVE being diagnosed and acquired within one year following the valvular replacement and late PVE occurring more than one year after replacement. If early PVE is received within the first days or weeks following replacement surgery, it is typically due to direct intraoperative contamination or hematogenous spread. When early PVE develops within two to 12 months, it is generally caused by a community-acquired or late-onset hospital-acquired infection. The organisms primarily responsible for early PVE within the first two months are *Staphylococcus aureus*, coagulase-negative staphylococci (most often *Staphylococcus epidermidis*), Gram-negative bacilli, and *Candida *species. The causal organisms of early PVE after the first two months are most often streptococci, *Staphylococcus aureus*, and coagulase-negative staphylococci, followed by enterococci. Aside from these, culture-negative organisms, fungi, and viruses may also occur in early and late PVE [7].

*Kocuria kristinae* has traditionally been viewed as a low-virulence microorganism prevalent in skin flora and oral mucosa. Categorized initially under the genus Micrococcus, it was considered a benign component of normal skin microbiota. However, a reclassification under the genus *Kocuria *has provided more insight into its characteristics. This Gram-positive coccus exhibits catalase positivity, coagulase negativity, and DNAase negativity and functions as a facultative anaerobe. A notable distinguishing feature of *Kocuria *lies in its sensitivity to bacitracin and lysozymes, whereas staphylococci are resistant to these agents. While historically linked to immunocompromised individuals and intravenous drug users, recent studies indicate a more comprehensive range of affected patients. Risk factors associated with *Kocuria *infections include chronic and subacute catheterization and patients with end-stage renal disease on peritoneal dialysis [8]. *Kocuria *biofilms are suspected but have not been demonstrated; our patient had no obvious culprit for his third presentation of IE. His previously infected valve had been replaced seven months prior, and he had no other indwelling non-native material besides the new bioprosthetic valve. It is possible that *Kocuria’s *antibiotic resistance played a role in our patient’s presentation.

In a comprehensive systematic review conducted by Napolitani et al. in 2018-2019, 48 cases of various *Kocuria kristinae* infections were identified, 20 of which met specific criteria and were selected for detailed analysis. Among these, there were 11 cases of catheter-associated bacteremia, four of infective endocarditis, and five of intra-abdominal infections. The IE cases presented a diverse clinical spectrum, including a 74-year-old man with a diabetic foot infection, a 56-year-old man with native valve endocarditis, a 35-year-old man with chronic hepatitis C linked to intravenous drug abuse who presented with a cerebrovascular accident secondary to his IE, and an 89-year-old woman with an infuse-a-port who was status post colectomy for ischemic bowel disease. Napolitani et al. theorized that *Kocuria kristinae* might pose a new challenge to public health due to its increasing prevalence, development of antibiotic resistance, and biofilm association. Subsequent case reports have supported this theory, emphasizing the emerging nature of *Kocuria kristinae* as a pathogen that demands vigilant monitoring and prompt intervention.

The virulence of *Kocuria kristinae* lies in its ability to cause clinically significant infections, challenging the perception of its historical insignificance. The management and identification of *Kocuria kristinae* infections pose distinctive challenges, necessitating a nuanced approach. In clinical practice, identification of bacteremia typically involves microbiological techniques such as standard blood cultures. However, *Kocuria kristinae* is often challenging to isolate, and accurate species identification requires advanced molecular methods, such as 16S rRNA and matrix-assisted laser desorption/ionization time-of-flight mass spectrometry (MALDI-TOF-MS), not typically available at most hospital laboratories [8]. *Kocuria *is morphologically similar to staphylococci and, as noted, has been generally regarded as a contaminant rather than a causative agent and is often misidentified [8]. In our patient’s case, it was initially suspected to be a contaminant rather than a source of bacteremia until TEE confirmed the heart valve vegetation. Due to its facultative anaerobic nature and characteristic sensitivity to bacitracin and lysozyme, differential diagnostics from other coagulase-negative staphylococci are crucial. Antimicrobial susceptibility testing is essential for guiding appropriate treatment, with reports suggesting susceptibility to various antibiotics, including vancomycin, linezolid, and ciprofloxacin, although resistance patterns may vary [8]. 

## Conclusions

The evolving recognition of *Kocuria kristinae* as a causative agent in IE presents a complex and clinically challenging scenario. Once considered a non-pathogenic microorganism of low virulence primarily associated with skin flora, recent evidence challenges this perception, revealing a broader spectrum of superficial and invasive Kocuria infections in individuals across diverse clinical scenarios. The variability in clinical presentations, coupled with the organism's capacity to induce IE even in immunocompetent individuals lacking traditional risk factors, underscores its emerging pathogenic potential. Clinicians should be vigilant in considering *Kocuria kristinae* as a potential pathogen in suspected bacteremia and IE and adapt identification and treatment strategies accordingly. Given the organism's emerging nature, continual surveillance and research are essential for refining identification methods, understanding antibiotic resistance patterns, and optimizing therapeutic approaches. 
